# Modelling and fitting the Polaron Pair Magnetoconductance model to obtain a realistic local hyperfine field in Tris-(8-hydroxyquinoline)aluminium based diodes

**DOI:** 10.1038/s41598-019-40132-5

**Published:** 2019-03-05

**Authors:** Zhichao Weng, William P. Gillin, Theo Kreouzis

**Affiliations:** Materials Research Institute and School of Physics and Astronomy, Queen Mary University of London, Mile End Road, E1 4NS London, United Kingdom

## Abstract

The Polaron Pair (PP) model has been successfully applied to magnetoconductance (MC) in organic semiconductor devices under ultra-small magnetic fields (USMFE). We report µT resolution MC measurements carried out with high sensitivity (better than 10^−6^) on the common organic semiconductor *tris*-(8-hydroxyquinoline)aluminium in the range ±500 µT displaying clear minima at ~±240 µT. Unlike traditional approaches, where device MC is simply evaluated using the PP model using nominal parameters for microscopic quantities such as the local hyperfine magnetic field, we have carried out actual fitting of the PP MC model to the experimentally obtained data. The fitting procedure yields physically realistic values for the polaron pair decay rate, local hyperfine magnetic field and triplet contribution to dissociation namely: $k$ = 28.6 ± 9.7 MHz, ${B}_{hf}$ = 0.34 ± 0.04 mT and ${\delta }_{TS}$ = 0.99 ± 0.01 respectively. The local hyperfine field obtained by fitting is in excellent agreement with independently calculated values for this system and is reproducible across different devices and independent of drive conditions. This demonstrates the applicability of the fitting approach to any organic USMFE MC data for obtaining microscopic parameter values.

## Introduction

Organic magnetoconductance (MC) is the change in device conductance when exposed to an external magnetic field. MC and its counterpart, organic magnetoresistance (MR), have been studied over the last decade and there are different theoretical models describing the effect^1–6^. Different models apply under different experimental conditions and it is often difficult to distinguish which models best describe experimental observations. Conventional MC measurements are performed at magnetic fields from tens to hundreds of mT, which greatly exceed the hyperfine fields present in organic materials^2^. Interesting phenomena emerge when the applied field is reduced to magnitudes comparable to or smaller than typical hyperfine fields in organic materials, i.e. smaller than a few mT. At such small fields the effect on conductance is measurable and is termed Ultra-Small Magnetic Field Effect (USMFE), in contrast to more commonly measured High Field Effects (HFE). Historically, small magnitude magnetic field effects were related to the recombination of radical ions in chemical reactions^7^. The effect of an externally applied field was theoretically related to the probability of forming singlet state radical ion pairs (singlet yield) and this tends to a different asymptotic value depending on magnetic field. The theory was extended to small applied fields and the low field effect on radical ion pairs has been studied, experimentally and theoretically^8,9^. Initial work led to a later radical pair model, used to successfully explain the small magnetic field behaviour of radical ion pair recombination in chemical reactions^10,11^ including elucidating the mechanism for avian navigation in weak (geomagnetic) fields^12,13^. In 2008, F. J. Wang *et al*. reported low field MC measurements on a hole-only organic semiconductor diode at 100 K. The low field MC displayed a sign reversal compared to the large field MC, yielding a characteristic “W” shaped MC when plotted versus applied magnetic field^14^. This initial USMFE report was followed by several studies^15–26^ including work by Nguyen *et al*. on a deuterated organic system below 1 mT. A theoretical model related to the radical pair model, termed the polaron pair (PP) model^15^ has been developed, where the singlet (or triplet) polaron pair yield is magnetically affected. Here we study the small applied field MC on a common organic semiconductor, *tris*-(8-hydroxyquinoline)aluminium (Alq_3_), and obtain typical W-shaped USMFE MC below 500 μΤ. We use the MC measured to demonstrate successful PP model fitting to experimental data, returning physically significant parameters, such as the local hyperfine field strength, ${B}_{hf}$.

Historically^15,16,19–24^ the PP model has been modified to include more complex interactions and more than one hyperfine field contributions^22^. There have been several instances of the MC resulting from these models being plotted, using representative *B*_*hf*_ values between 1 and 5 mT^20–22,24^, which correspond to the fields obtained by empirical (usually Lorentzian) fitting^27–29^ of experimental MCs. These traditional approaches have been successful in reproducing the functional forms of experimentally obtained MC data for a number of systems^15,20–22,24^, but they are based on calculating the MC resulting from PP models using historically reported or “typical” hyperfine field values for organic systems. This differs fundamentally from our approach where PP model fitting is carried out on MC data with no assumptions regarding microscopic parameter values such as the (average local) hyperfine field experienced by the polaron.

We have obtained sufficiently high quality data to perform fitting of the PP model to experimental MC data and obtain model parameters with associated uncertainties. The fitting has been carried out on data obtained from several devices and over a range of drive conditions and returns consistent fitting parameters within error (see Supplementary Material S2). This raises the exciting prospect that PP model fitting to experimental MC data can be used more generally as a method of obtaining microscopic parameters (e.g. average local *B*_*hf*_) in a variety of organic systems.

## Results

**Figure 1:**
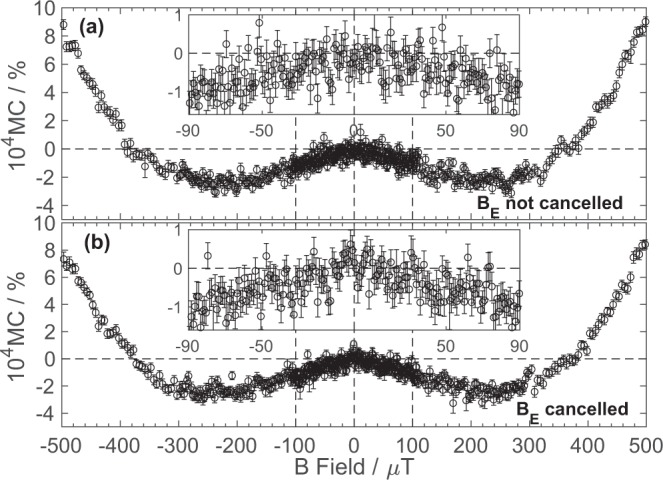
(**a**) Room temperature Ultra Small Magnetic Field Magnetoconductance measured in a standard Alq_3_:NPB device in the presence of the Earth’s magnetic field. The inset displays the averaged MC obtained at low applied fields. (**b**) Room temperature Ultra Small Magnetic Field Magnetoconductance measured in the same device with the Earth’s magnetic field externally cancelled. The inset displays the averaged MC obtained at low applied fields. All the error bars shown represent the standard error for each data point as defined in Supplementary Information S4.

Figure 1 displays the USMFE MC results obtained from a device under 2 µA constant current in the presence and absence of the Earth’s magnetic field (i.e. nulled in the two orthogonal directions to the varied field). In both cases the results show a minimum MC magnetic field (*B*_*m*_) at approximately ±240 µT and 2 × 10^−4^% magnitude. The standard error for each data point is 3.6 × 10^−5^% (see Supplementary Information S4 for details) and the two plots are essentially identical above 100 µT. The insets show the MC below 100 µT. The two data sets only differ slightly for applied fields below ~45 µT, i.e. for fields smaller or equal to the vector sum of the components of Earth’s magnetic field orthogonal to the applied field direction. In the presence of the Earth’s field, the sample shows little MC below 45 µT in contrast to Fig. 1b (inset) where a slightly steeper, but noisier, MC is obtained. This is expected since the external field is applied in an arbitrary direction (in this case horizontally at a bearing of 60°) and we should obtain measurable effects once the externally applied field exceeds the component of Earth’s field that has not been nulled. We do not notice any “shifts” of the MC response, in contrast to some literature results^16^, where measurements were deliberately carried out parallel and antiparallel to the Earth’s field. Again, our results are as one would expect as we are plotting the total *measured* field experienced by the device and hence the external field outside the coils would not be expected to affect the device. Figure 1b demonstrates that it is possible to carry out µT resolution MC measurements at fields smaller than the Earth’s field with a MC sensitivity below 10^−6^ (1 ppm, 10^−4^%), using external field cancelling.

**Figure 2:**
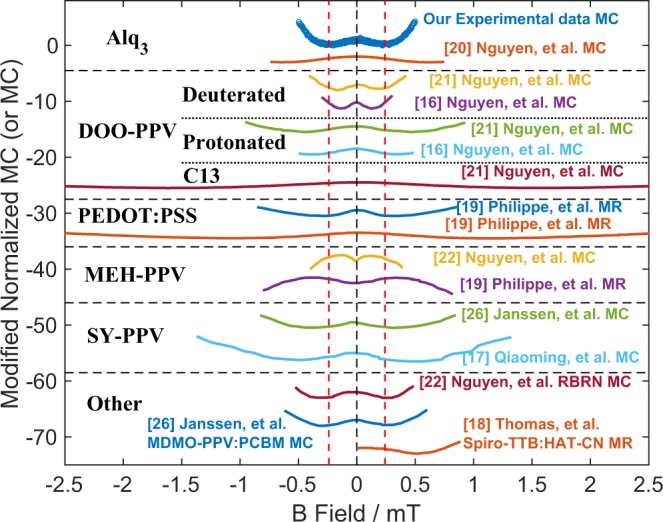
Normalised MC (or MR) versus magnetic field in a variety of samples (including this study and from literature^16–22,26^). The data have been normalised using Equation (1) and the different datasets have been vertically displaced for clarity. The sources and main materials studied are indicated in the plot. The red dashed vertical lines indicate *B*_*m*_ in our sample.

To place our results in context we show normalised MC (or MR) for a variety of different organic materials, including ours, and from refs^16–22,26^, in Fig. 2. The normalisation was carried out using Equation (1).

1 $$M{C}_{normalised}=\frac{MC(B)}{MC(B={B}_{m})}$$

**Figure 3:**
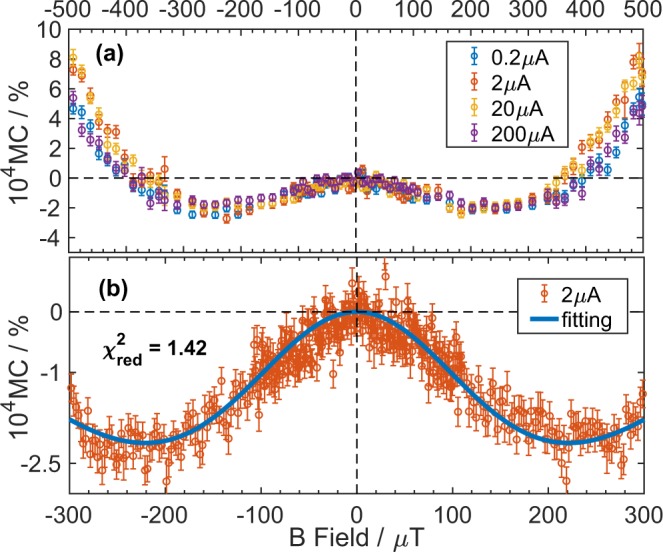
(**a**) Averaged experimentally obtained MC results (having cancelled the geomagnetic field using external coils) with different drive current. The large field (above approximately 400 µT) results show some dependence on drive current. (**b**) The 2 µA drive current MC results in the range ±300 µT. The solid line is a fit obtained using the polaron pair model, Equations (2–7) and the parameter values:*k* = 28.6 ± 9.7 MHz, *B*_*hf*_ = 0.34 ± 0.04 mT and ${\delta }_{TS}$ = 0.99 ± 0.01. The ${\chi }_{red}^{2}$ returned is 1.42. All the error bars shown represent the standard error for each data point as defined in Supplementary Information S4.

Where *MC(B*) is the MC at a given field and *MC(B* = *B*_*m*_*)* is the MC at the magnetic field where it reaches its minimum (or maximum) value, *B*_*m*_. Our data yield $|{B}_{m}|\approx$ 240 µT, placing the results within the USMFE range. Note that there are variations in USMFE results, even for the same (nominal) system, for example in the H-DOO-PPV results of Nguyen and co-workers (refs^16,21^), as well as variations in MC for the same system between different groups, e.g. our Alq_3_ results compared to ref.^20^. The discrepancies between our experimental MC in Alq_3_ and those of ref.^20^ could be due to a number of factors: different device architectures, drive conditions, magnetic field range and instrumental resolution. In any case, they are not addressed further as they fall outside the scope of this article. We also note that the smallest literature ${B}_{m}$ fields are displayed by deuterated samples^16,21^, as expected given that deuterated samples display smaller hyperfine magnetic fields compared to protonated samples. Measurement conditions and architectures (unipolar, ambipolar) can also yield variations in USMFE results as demonstrated by the MEH-PPV MC of refs^19,22^. By controlling the drive conditions in our devices, we ensure stable and reproducible USMFE MC results, for example comparing datasets in Figs 1 and 2, obtained several months apart on the same device. Additionally the device drive current does not affect the USMFE MC obtained (below ~300 µT, Fig. 3). Apart from deuterated samples, the $|{B}_{m}|\approx$ 240 µT is at the lower end of the protonated literature results. Given the high magnetic field resolution, the high MC sensitivity and the availability of a quantified error for each MC data point obtained by our experimental setup, the datasets obtained are ideal for the purpose of fitting using a suitable theoretical model.

The approach we take is to apply the PP model as the basis for fitting the MC obtained. This approach is not limited to our own experimental data, but in principle can be applied to any MC results with a suitably defined MC. The PP model has been applied to organic devices in the past^15,20–25^ and is based on a magnetic field dependent singlet-triplet interconversion. Singlet or triplet polaron pairs interconvert into each other over time and a magnetic field affects this behaviour, determining the ultimate singlet and triplet polaron pair yields. The polaron pair, an electron-hole pair bound within a Coulomb radius, can either combine to form a tightly bound exciton or dissociate back into free charges. Since singlet excitons decay radiatively, magnetically induced changes in the singlet exciton yield will appear as electroluminescence. In parallel, since singlet and triplet polaron pairs contribute differently to dissociated carriers, any changes in singlet (or triplet) yield will result in a different number of free carriers due to dissociation. These will change the overall carrier density and result in MC.

The approach taken by Timmel *et al*.^11^. was to initially assume the formation of singlet polaron pairs (this is arbitrary and the same physics can be obtained by assuming initial triplet formation) with initial singlet density, $\sigma (0)$, and then calculate the time and magnetic field dependent spin density, $\sigma (t)$, using Equation (2).

2 $$\sigma (t)={e}^{-i {\mathcal H} t}\sigma (0){e}^{i {\mathcal H} t}$$

Where ${\mathcal H}$ is the spin Hamiltonian describing the interactions between the polaron pair and the external magnetic field. In general, the Hamiltonian is expected to contain the Zeeman interaction between the polaron and the external magnetic field, the hyperfine interaction between the polaron and the hydrogen nuclei, dipolar interactions and exchange interactions between the spins of each polaron etc. For simplicity a reduced, one proton, Hamiltonian is used containing only the Zeeman and hyperfine interactions, shown in Equation (3).

3 $$ {\mathcal H} =g{\mu }_{B}{S}_{1z}B+g{\mu }_{B}{S}_{2z}B+g{\mu }_{B}{B}_{hfc1}{{\boldsymbol{S}}}_{1}\cdot {\boldsymbol{I}}$$

Where $g$ is the g-factor, *µ*_*B*_ is the Bohr magneton, *B* is the applied magnetic field, *S*_1*z*_ and *S*_2*z*_ are the *z* components of the spin operators for the two polarons and *B*_*hfc1*_ is the local hyperfine field due to a single proton. ***S***_1_ is the spin operator including all components for one polaron and ***I*** is the spin operator for the hydrogen nucleus. The first two terms of Equation (3) correspond to the Zeeman interaction, while the third term represents the hyperfine interaction.

To calculate the singlet fraction, ${\rho }_{s}$, the trace of the singlet projection operator, ${P}_{s}$, on $\sigma (t)$, as shown in Equation (4), is required.

4 $${\rho }_{s}={\rm{Tr}}[{P}_{s}\sigma (t)].$$

We have used a specific form of the singlet projection operator in Equation (5) which differs from the generalised operator appearing in ref.^11^.

5 $${P}_{s}=\frac{1}{4}\times {I}_{e8\times 8}-{I}_{Ax}\otimes {I}_{Bx}\otimes {I}_{e2\times 2}-{I}_{Ay}\otimes {I}_{By}\otimes {I}_{e2\times 2}-{I}_{Az}\otimes {I}_{Bz}\otimes {I}_{e2\times 2}$$

Where ${I}_{e8\times 8}$ is an 8 × 8 unity matrix and *I*_*A*_ and *I*_*B*_, subscript *x*, *y* and *z*, are the corresponding components of the Pauli matrices of each polaron. For a detailed description of the calculation, see Supplementary Information S5. The effect of the singlet projection operator as used in Equation (4) is to “filter out” all the singlet components among all spin configurations.

Finally, the steady state singlet yield, ${{\rm{\Phi }}}_{s}$, is obtained by integrating the singlet fraction over all time, assuming a single rate constant, $k$, to account for the disappearance of the singlets by various mechanisms, using Equation (6).

6 $${{\rm{\Phi }}}_{s}=k{\int }_{0}^{\infty }{\rho }_{s}(t){e}^{-kt}dt.$$

According to the literature^11^, spin selective radical-radical reactions (in our case, polaron-polaron interactions, such as the dissociation or recombination of polaron pairs) can occur, resulting in the disappearance of singlet and triplet excited states. Thus, the corresponding fractions decay with time and can be described by first-order kinetics. The polaron-polaron interaction occurs for both singlet and triplet polaron pairs, however, although they possess different spin configurations the large inter-polaron distance can make the energy difference between the two relatively small. Additionally, the spin-dependent recombination kinetics become inter-twined with the spin-dependent coherent evolution^11^. Given that the scope of the present work is to report the technique of fitting the polaron pair model to experimentally obtained data and extracting physically significant parameters, the use of a single decay rate is further justified by reducing the total number of fitting parameters used.

By using different values for the applied magnetic field throughout the calculation, we obtain the magnetic field dependent singlet yield, ${{\rm{\Phi }}}_{s}(B)$. From this the triplet yield, ${{\rm{\Phi }}}_{T}(B),$ can be evaluated simply, using ${{\rm{\Phi }}}_{s}(B)+{{\rm{\Phi }}}_{T}(B)=1$. Similarly to Nguyen and co-workers^21^ the yields are used to evaluate the MC at a given field using Equation (7).

7 $$MC(B)=\frac{{{\rm{\Phi }}}_{s}(B)+{\delta }_{TS}{{\rm{\Phi }}}_{T}(B)}{{{\rm{\Phi }}}_{s}(B=0)+{\delta }_{TS}{{\rm{\Phi }}}_{T}(B=0)}-1.$$

We note that making a direct link between the magnetic field dependent singlet (or triplet) yield and the MEL is straightforward since only singlet states are emissive (for example, see ref.^15^), but the relationship between the yields and the resultant MC is not as simple. We have used Equation (7) to relate yields to MC and ultimately model our experimental data, as it has been shown to be successful in the literature^21^, but we offer no more detailed a mechanism than to simply state that different polaron pair dissociation (singlet, triplet) will alter the number of free charge carriers contributing to conduction. The significance of the dimensionless factor, ${\delta }_{TS},\,\,$in Equation (7) is to describe the relative contributions of singlet and triplet polaron pairs to conduction via dissociation, offering a microscopic mechanism for MC.

Our approach is to use a single, average, local hyperfine field (single proton modelling) unlike approaches where two or more proton fields are considered. This is because we are solely investigating the application of fitting a data set to the model to extract parameters, with their associated uncertainties.

Using the approach outlined in Equations (2–7) we can fit the experimentally obtained $MC(B)$ data using just three fitting parameters, namely: $k$, ${B}_{hf}$ and ${\delta }_{TS}$. The quality of the fit is quantified using a reduced, normalised chi-squared value, ${\chi }_{red}^{2}$ (see Supplementary Information S6 for a detailed description of the fitting procedure).

In practice, a 200 by 200 by 200 matrix of ${\chi }_{red}^{2}$ values is constructed using 200 different values for each free fitting parameter ($k$, ${B}_{hf}$ and ${\delta }_{TS}$), with the lowest ${\chi }_{red}^{2}$ yielding the best fit variable values (see Supplementary Information S6). In order to obtain errors for the fitting parameters returned by the procedure, we recorded the range of each parameter within one standard error of the minimum ${\chi }_{red}^{2}$ value, ${\sigma }_{{\chi }_{red}^{2}},$ given by: ${\sigma }_{{\chi }_{red}^{2}}=\sqrt{2/N}$ where *N* is the number of data points.

Figure 3a shows the MC obtained from one sample at room temperature using different current drive conditions with the geomagnetic field cancelled. We note that the USMFE MC does not depend on drive current (below approximately 300 µT), consistent with the PP model^11^, and deviations between data sets only occur at large fields where one expects different microscopic mechanisms to apply (for example, see ref.^30^). Since the USMFE MC does not depend on drive conditions within the range studied, we fit the PP model to the 2 µA current data set shown in the Fig. 3b.

The minimum ${\chi }_{red}^{2}$ set of parameters obtained by the fit in Fig. 3b are:

$k$ = 28.6 ± 9.7 MHz, ${B}_{hf}$ = 0.34 ± 0.04 mT and ${\delta }_{TS}$ = 0.99 ± 0.01, with a ${\chi }_{red}^{2}$ = 1.42.

## Discussion

At this point we must address the parameter values obtained by fitting and their physical significance. We begin by considering the disappearance rate constant, $k$. This is consistent with the value of ${B}_{hf}$, since the rate constant has to be smaller than the Larmor frequency for the corresponding hyperfine field, which in this case is 59 MHz^11^. The local ${B}_{hf}$ of 0.34 mT, is much smaller than typical hyperfine fields quoted in the literature^27–29^, but is comparable to local hyperfine fields calculated by Marumoto *et al*. using Density Functional Theory (DFT) for an Alq_3_ anion^31^ where different local hyperfine field magnitudes between 0.01 mT and 1.43 mT are reported.

In trying to assess the relevant local hyperfine field experienced by a polaron one has to take into account the spatial distribution and location of the Highest Occupied Molecular Orbital (HOMO) and Lowest Unoccupied Molecular Orbital (LUMO) wavefunctions in Alq_3_. Using available literature calculations^32,33^ for the spatial distribution of the HOMO and LUMO in our system we are able to calculate the average local hyperfine field in each case using the relevant literature numerical values^31^ and averaging methods^25^. Thus we obtain an average local hyperfine field value for the HOMO of approximately 200 µT and for the LUMO of approximately 1.8 mT. Our ${B}_{hf}$ value of 0.34 mT therefore appears to correspond to the average local field for the HOMO and thus should correspond to the field experienced by the hole in the polaron pair and not the electron. Since the data obtained by us is limited to small (100 s of µT) fields, we would not expect to detect large hyperfine field component contributions, such as those resulting from the electron (LUMO) average local hyperfine fields. In terms of the single proton model applied by us, the choice is entirely justified as in this model only one of the polaron pair charges is coupled to the hyperfine field, which in our case is the positive (hole) polaron. Furthermore, our *B*_*hf*_ value of 0.34 mT can be compared to local fields measured by an entirely independent method. Drew *et al*. have obtained neighbouring proton nuclear spins coupling to electrons, using muon spin relaxation measurements, below approximately 0.3 mT^34^ in Alq_3_. Thus, the local *B*_*hf*_ value returned by the Polaron Pair theoretical fit to our data is in excellent agreement with relevant local hyperfine fields calculated, for this molecule, entirely independently by two different methods. Additionally, Electron Spin Resonance spectroscopy has been used to measure the local hyperfine field in the organic semiconductor H-DOO-PPV and returned a value of 0.37 mT (which compares favourably to our fitted *B*_*hf*_ value for Alq_3_)^25^. We note that “hyperfine” fields of order 3–5 mT reported in literature^27–29^ for different organic semiconductors, including Alq_3_, are obtained using empirical (usually Lorentzian) fits to MC data, but some empirical line-shapes, such as those used by Janssen *et al*.^26^., do yield “hyperfine” fields below 1 mT.

The ${\delta }_{TS}$ value of 0.99 indicates that triplet polaron pairs contribute less to dissociation than singlet polaron pairs, in agreement with ref.^21^ who also report a ${\delta }_{TS}$ of less than one. This is physically reasonable, since triplet polaron pairs can be expected to be more strongly bound than singlets. This is certainly true of triplet versus singlet excitons^35^ and a more strongly bound pair is expected to have a smaller probability of dissociation. The microscopic parameters obtained by fitting the sample results in Fig. 3 are consistent (within error) with those obtained from different diodes measured at the same drive current and with the parameters obtained from a single sample at drive currents between 0.2 µA and 200 µA (see Supplementary Material section S2). Additionally, there was no evidence of device degradation, despite the large number of repetitions, as evidenced by comparing the MC obtained over different numbers of repetitions, at the beginning and end of a given experiment, and over all averages shown in Supplementary Information S2. Notably, a small local *B*_*hf*_ of ~300 µT is always obtained, vindicating the approach of modelling a single average local hyperfine field. It should be stressed that the PP model plots appearing in the literature^22^ using larger (1 mT, 3 mT) two proton fields are not fits to data and are used solely as demonstrations that the model can reproduce the correct “W” MC shape, that is, the functional form of the MC.

In conclusion, we report USMFE MC measurements in Alq_3_ based devices with µT resolution and ppm sensitivity at fields comparable to the Earth’s magnetic field (with careful external coil cancellation). The MC measured displays one of the smallest minimum field values (${B}_{m}$ ~ 240 µT) reported for protonated organic systems and is not a function of drive current (unlike the HFE MC measured). The data has been successfully fitted using the Polaron Pair model for organic magnetoconductance and returns physically significant values for three fitting parameters: $k$ = 28.6 ± 9.7 MHz, ${B}_{hf}$ = 0.34 ± 0.04 mT and ${\delta }_{TS}$ = 0.99 ± 0.01. The parameter values are mutually consistent, and the average local hyperfine field value of 340 µT obtained by us for Alq_3_ agrees with literature DFT modelling and muon based measurements for this material. Additionally, it is considerably smaller than the Lorentzian empirical fit values in the range 1–5 mT reported in the literature for organic systems. The ~340 µT value of *B*_*hf*_ obtained by us for Alq_3_ does not depend on individual device drive conditions and is reproducible across different devices, indicating it represents an actual microscopic material property, within the context of the PP model, rather than depending on individual experimental conditions. Thus, our report demonstrates that Polaron Pair based theoretical fitting of experimentally obtained MC data can be a viable method of obtaining values for physically significant microscopic quantities such as *B*_*hf*_ in any organic system.

## Methods

### Device Fabrication

The overall device structure used consisted of an indium tin oxide (ITO) anode, a N,N′-Di(1-naphthyl)-N,N′-diphenyl-(1,1′-biphenyl)-4,4′-diamine (NPB) hole transport layer, an Alq_3_ electron transport/emission layer, a LiF electron injection layer and finally an aluminium cathode, typically: ITO/NPB(50 nm)/Alq_3_(50 nm)/LiF(1.5 nm)/Al(100 nm) and 4 mm^2^ individual diode area. NPB and Alq_3_ were purchased from Sigma-Aldrich Inc. and purified twice using train sublimation before use. Patterned ITO coated glass substrates were used for diode fabrication with all subsequent layers deposited by vacuum deposition (evaporation). Typical parameters used were: 10^−7^ mbar base pressure, ~0.2 nm·s^−1^ NPB and Alq_3_ deposition rates, 0.02 nm·s^−1^ LiF deposition rate, 0.06 nm·s^−1^ for the initial 10 nm and 0.5 nm·s^−1^ thereafter for Al deposition.

### Magnetic field effect measurement and analysis

A 3-D Helmholtz coil system was used both to cancel the Earth’s magnetic field and provide the applied field for the MC measurements (for details see Supplementary Informationl S1). The applied field coils were driven by a Keithley 2400 SourceMeter unit while the current through the diode was provided by an Agilent B2902A source-measure unit, the voltage across the device was measured using a Keithley 4200 semiconductor characterisation system. Magnetic field measurements were made using a LakeShore 475 DSP Gaussmeter and all equipment was controlled via GPIB using custom written software. The B values plotted are the actual B-fields recorded by the gaussmeter at each data point. Typical B-field step sizes are ~1 µT, 2.5 µT and 5 µT in the B field regimes of ±100 µT, ±(100–300)µT and ±(300–500) µT, respectively. The device voltage at different fields was recorded under constant current and readings were repeated 100 times for averaging. Voltage measurements with applied field,$\,V(B)$, were alternated with zero field measurements, $V(0)$, to eliminate device drift by averaging the two zero field readings (before and after). The MC was calculated using Equation (8).

8 $$MC=\frac{V(0)-V(B)}{V(B)}\times 100 \% $$

All measurements were carried out with the diode under vacuum (10^−5^ mbar) at room temperature. We note that no measurable device degradation was observed, despite hundreds of measurement repetitions (see Supplementary Information S2 for details).

## Supplementary information

Supplementary information

## Data Availability

The datasets generated during and/or analysed during the current study are available from the corresponding author on reasonable request.
