# Supplementary material for: Modelling and fitting the Polaron Pair Magnetoconductance model to obtain a realistic local hyperfine field in Tris-(8-hydroxyquinoline)aluminium based diodes
Source: Sci Rep. 2019 Mar 5;9:3439. doi: 10.1038/s41598-019-40132-5 (PMC6401170; doi:10.1038/s41598-019-40132-5)
Supplement: Supplementary file 1 — Supplementary information [file 41598_2019_40132_MOESM1_ESM.pdf]

Supplementary Information

Modelling and fitting the Polaron Pair Magnetoconductance model to obtain a realistic local hyperfine field in Tris-(8-hydroxyquinoline)aluminium based diodes

Zhichao Weng, William P. Gillin and Theo Kreouzis*

Materials Research Institute and School of Physics and Astronomy, Queen Mary University of London, Mile End Road, E1 4NS, London, United Kingdom.

* t.kreouzis@qmul.ac.uk

S1 Experimental set-up and device structure

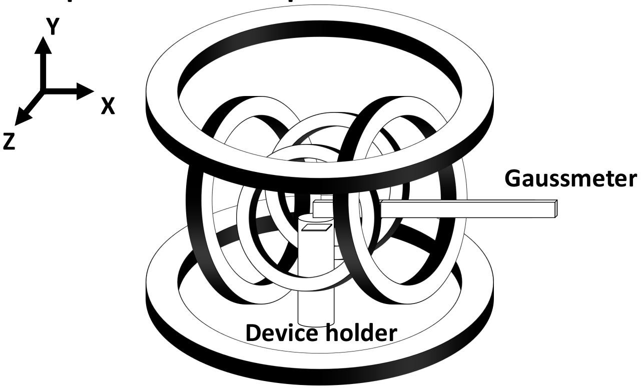

Figure S1-1. Schematic of the 3-Dimensional Helmholtz coil system

The system consists of 3 pairs of orthogonal coils of different sizes oriented in the x, y and z directions (referred to as x, y and z coil pairs). The sample was mounted in a sample holder, located at the center of the system. The homogeneity of the magnetic field generated by the coil across the device was measured and is shown in Figure S1-2.

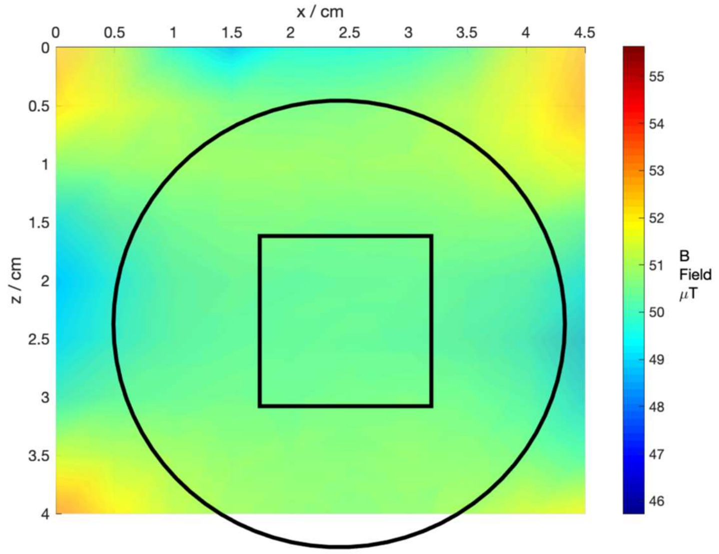

Figure S1-2. Homogeneity of the magnetic field along XZ plane.

In Figure S1-2, the circle region is where the device holder is and the 1.5 x 1.5 cm square region is where our device lies. The actual diode is a 2 x 2 mm region within this square. In order to measure this homogeneity, the gaussmeter was first placed in the centre of the square and the Z coil current adjusted to produce a B field of 51 μT at that point. With the coil current fixed we moved the gaussmeter to pre-mapped coordinates to map the magnetic field across the region. The average B field across the device is 51.1μT±0.2μT.

The whole coil setup and the device were located in a position where the Earth magnetic field components along the x, y and z directions were measured to be approximately 14μT, 42μT and 10μT respectively. The magnetic field along the z direction was chosen as the scanning B field and the x and y applied DC fields simply used to cancel the Earth's field components. In this example, the x and y coils were connected to a DC power supply (Siglent SPD3303X) while the z coils were connected to a source measure unit (Keithley 2400 SourceMeter).

The sample holder is designed with no ferromagnetic components (for example, with custom made phosphor-bronze springs in the sample contacts) which could provide stray fields or result in sample movement as the external field is switched.

The device structure is shown in Figure S1-3. And the fabrication processes and parameters are described in the main text.

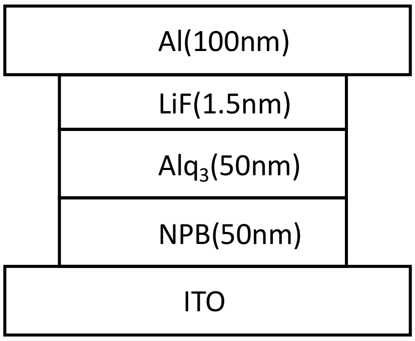

Figure S1-3. Schematic of device structure

S2 Reproducibility of MC results and the model fitting

In total, 3 devices based on the same structure shown in Figure S1-3 have been made and measured. The results are shown in Figure S2-1.

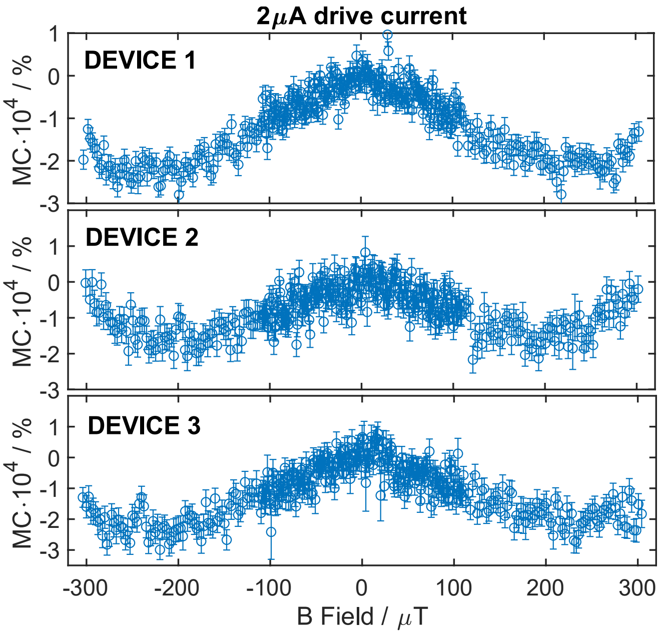

Figure S2-1. MC results of 3 individual devices

Noticeably, the device used to produce all the MC results in the main text has not undergone any visible degradation, that can be observed, in the MC results. As shown in Figure S2-2, there are a total of 240 measurements taken to yield the total averaged dataset as shown in the manuscript and this whole measurement takes ~120 hours to complete. In order to see the effect of any possible degradation on the MC, we took the first and last 10 measurements from our 240 measurement dataset and averaged each of them. As can be seen in Figure S2-2, there is no significant difference between the first 10 and last 10 averaged datasets or with the average of all 240 (with the exception of the noise level), and this demonstrates that the device behaves in a reproducible manner at the very beginning of the measurements and after hundred hours of measuring.

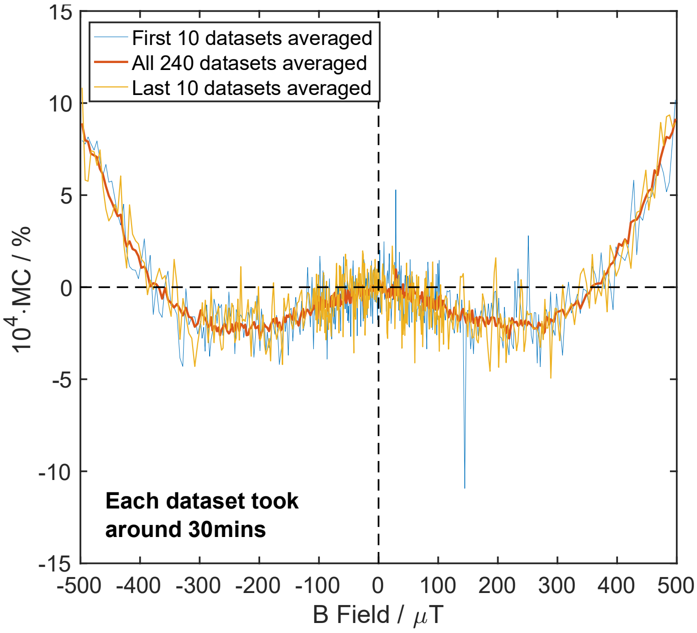

Figure S2-2. Datasets for first and last 10 measurements compared to the whole measurements

To show the reproducibility of our model fitting, we used the approach described in the main text to fit each individual device and the results are shown in Table S2-1. From the results, all the parameters are consistent with each other within error bars.

Table S2-1. Fitting parameters for 3 individual devices within ±300μT

	DEVICE 1 (Main text)	DEVICE 2	DEVICE 3
B_{hf}	**(0.34 ± 0.04) mT**	**(0.30 ± 0.03) mT**	**(0.34 ± 0.02) mT**
k	**(28.6 ± 9.7) MHz**	**(20.2 ± 2.6) MHz**	**(35.7 ± 2.2) MHz**
δ_{TS}	**0.99 ± 0.01**	**0.99 ± 0.01**	**0.98 ± 0.01**

Fitting was performed for each of the drive currents shown in Fig 3 in the main text and the results are shown in Table S2-2. In our model fitting, B_{hf} largely determines the horizontal positions (magnetic field) of the "W" shape MC while k determines the vertical position of the two dips (the magnitude of MC). For device 1, 240 averages were taken and the other two devices only 100 averages were taken for each, the errors in data of device 2 and 3 are therefore slightly larger than device 1. As our Helmholtz coils and gaussmeter are stable and precise there is little effect on the errors of horizontal positions of two dips (magnetic field). Therefore, the main error is largely in the vertical position of two dips (the magnitude of MC). Our P-P model is very sensitive to the change of data and this slight change in MC magnitudes due to different averaging would result in slightly different k's as shown in Table S2-1. This also shows the consistency and reproducibility of our model fitting, with all values within the errors. The drive condition of 2μA was chosen for the majority of our measurements as low drive condition (0.2μA) generates relatively noisier MC under 100 averaging and high drive condition (20μA and 200μA) can generate lower noise MC though, but with the potential to degrade the device faster. Therefore 100 averages were used for the drive conditions of 0.2μA, 20μA and 200μA. And 240 averages were used for 2μA drive condition to generate the best data for our model to fit. We have actually measured a single device for a total of ~7000 scans, with a total on-time of ~200 days and we observe the same fitting parameters for experiments on the aged device as on new ones.

Table S2-2 Fitting parameters for different drive conditions of device 1 within ±300μT.

	**0.2μA**	**2μA**	**20μA**	**200μA**
B_{hf}	**(0.32 ± 0.06) mT**	**(0.34 ± 0.04) mT**	**(0.32 ± 0.07) mT**	**(0.34 ± 0.07) mT**
k	**(20.7 ± 4.9) MHz**	**(28.6 ± 9.7) MHz**	**(22.2 ± 5.8) MHz**	**(24.5 ± 6.8) MHz**
δ_{TS}	**0.99 ± 0.01**	**0.99 ± 0.01**	**0.99 ± 0.01**	**0.99 ± 0.01**

S3 IV characteristic of the device

The IV measurement results are shown in Figure S3. Above 3V, the device is operating in ambipolar mode as evidenced by visible electroluminescence. The red dashed line at ~ 0.5 A $\cdot m^{-2}$ represents 2 μA drive current at around 3.4 V bias. The j-V is clearly superlinear in these conditions and the device is operating above any "turn on" threshold.

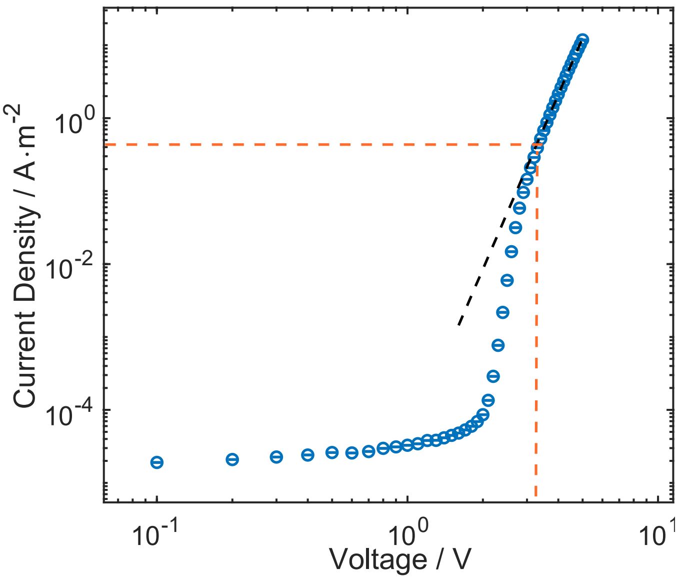

Figure S3 IV characteristic of the device

S4 Data mean values and error bars.

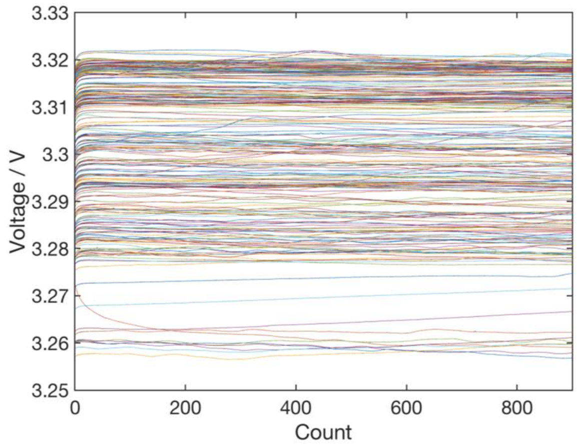

Figure S4-1. Constant current mode raw data of device voltage versus count. 100 repetitions of the acquisition are displayed under 2μA.

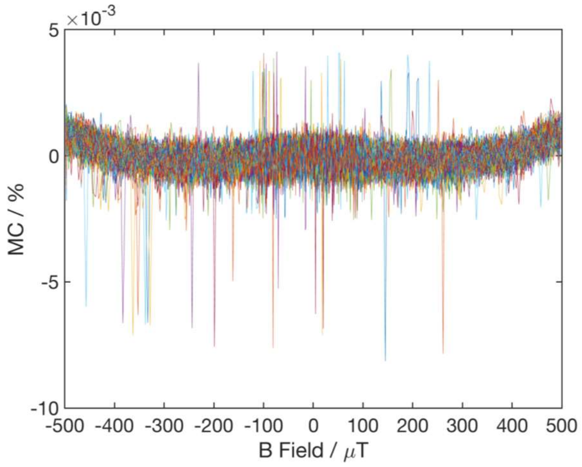

Figure S4-2. MC values calculated using equation (1) for each of the 100 repeated acquisitions.

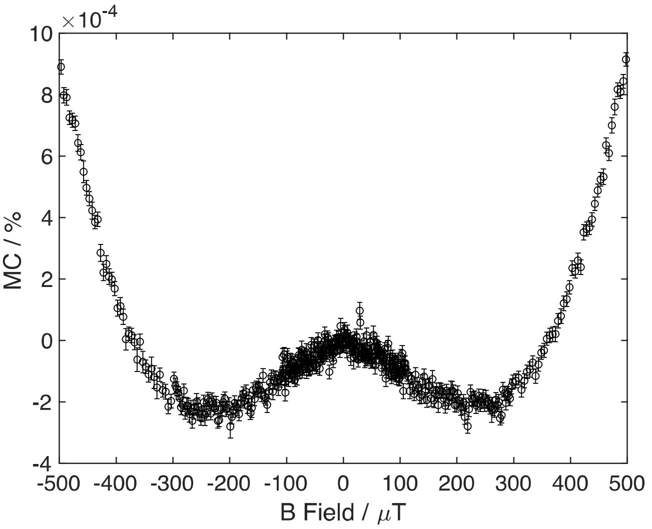

Figure S4-3. Resultant averaged MC, including the standard errors from 100 MC acquisitions.

The MC was measured when the diode was biased with constant current and the device voltage was recorded at each applied magnetic field. Figure S4-1 shows the raw data of device voltage plotted against the number of counts. The MC was measured under constant drive current and figure S4-1 represents the measured device voltage versus count, where the count represents any applied magnetic field and is always alternated with a null field measurement. Thus, the very small device drift, <2% over the whole measurement time, evident in figure S4-1 is eliminated from the MC calculation. We note that at 2 μA drive current there was no significant device degradation over hundreds of averages. Equation (1) was applied to calculate each MC value for the 100 repeated datasets (raw MC data is shown in Figure S4-2). The arithmetic mean and standard error were calculated from these 100 datasets using equations (SE1) and (SE2):

$$MC_{mean}(B) = \frac{1}{n}\sum_{n=1}^{100} MC_n(B) \qquad \text{(SE1)}$$

$$Standard\ Err. = \frac{\sigma}{\sqrt{n}} \qquad \text{(SE2)}$$

where $MC_n(B)$ is the MC value of the n-th repeated dataset at a specific applied magnetic field and σ is the standard deviation of each data point from the 100 experiments. The calculated MC_{mean} and $Standard\ Err.$ correspond to the MC values and error bars shown in Figure S4-3.

S5 Details of Polaron Pair model calculation

The spin density is calculated using the Liouville-von Neumann equation[11], equation (3).

$$\sigma(t) = e^{-i\mathcal{H}t}\sigma(0)e^{i\mathcal{H}t} \qquad (3)$$

Where $\sigma(0)$ is the initial spin density of the polaron pair system which is assumed to be a singlet initially, as explained in the main text. $\mathcal{H}$ is the spin Hamiltonian describing different interactions between polaron pairs and a single surrounding hydrogen nucleus, expressed in equation (4).

$$\mathcal{H} = g\mu_B S_{1z}B + g\mu_B S_{2z}B + g\mu_B B_{hfc1}\boldsymbol{S_1}\cdot\boldsymbol{I} \qquad (4)$$

where g is the g-factor, μ_B is the Bohr magneton, B is the applied magnetic field, S_{1z} and S_{2z} are the z components of the spin operators for the two polarons and B_{hfc1} is the local hyperfine field due to a single proton. $\boldsymbol{S_1}$ is the spin operator including all components for

one polaron of the pair and $\boldsymbol{I}$ is the spin operator for the hydrogen nucleus, defined in equation (SE5).

$$\boldsymbol{S_1} \cdot \boldsymbol{I_1} = \boldsymbol{S_{1x}} \cdot \boldsymbol{I_{1x}} + \boldsymbol{S_{1y}} \cdot \boldsymbol{I_{1y}} + \boldsymbol{S_{1z}} \cdot \boldsymbol{I_{1z}} \quad \text{(SE5)}$$

Physically, $\boldsymbol{S_1}$ and $\boldsymbol{I}$ with different x, y and z subscripts correspond to the Pauli matrices for the polaron and the hydrogen nucleus respectively.

Intuitively, the singlet-triplet basis matrix is defined as in equation (SE6).

$$P_e = \begin{pmatrix} 1 & 0 & 0 & 0 \\ 0 & 1/\sqrt{2} & 1/\sqrt{2} & 0 \\ 0 & -1/\sqrt{2} & 1/\sqrt{2} & 0 \\ 0 & 0 & 0 & 1 \end{pmatrix} \quad \text{(SE6)}$$

Where the second column represents singlet component while the other three columns represent three different triplet components. For the consistency of the quantum calculation, all the spin components should stay in the same singlet-triplet basis as shown in equation (SE6), such a transformation can be achieved using equation (SE7)[S1].

$$P = Pe \otimes I_e = \begin{pmatrix} 1 & 0 & 0 & 0 & 0 & 0 & 0 & 0 \\ 0 & 1 & 0 & 0 & 0 & 0 & 0 & 0 \\ 0 & 0 & 1/\sqrt{2} & 0 & 1/\sqrt{2} & 0 & 0 & 0 \\ 0 & 0 & 0 & 1/\sqrt{2} & 0 & 1/\sqrt{2} & 0 & 0 \\ 0 & 0 & -1/\sqrt{2} & 0 & 1/\sqrt{2} & 0 & 0 & 0 \\ 0 & 0 & 0 & -1/\sqrt{2} & 0 & 1/\sqrt{2} & 0 & 0 \\ 0 & 0 & 0 & 0 & 0 & 0 & 1 & 0 \\ 0 & 0 & 0 & 0 & 0 & 0 & 0 & 1 \end{pmatrix} \quad \text{(SE7)}$$

Thus, the singlet projection operator in the singlet-triplet basis can be calculated as shown in equation (6).

$$P_s = \frac{1}{4} \times I_{e\,8\times8} - I_{Ax} \otimes I_{Bx} \otimes I_{e\,2\times2} - I_{Ay} \otimes I_{By} \otimes I_{e\,2\times2} - I_{Az} \otimes I_{Bz} \otimes I_{e\,2\times2} \quad (6)$$

The singlet fraction and the magnetic field dependent singlet yield were calculated as described in the main text (equations (5) and (7)).

Notably, the final form of the singlet yield can be expressed in the form in equation (SE8)[11]:

$$\Phi_s = \frac{3}{8} + \frac{1}{8}\frac{\omega^2}{\Omega^2} + \frac{1}{8}\frac{a^2}{\Omega^2} f(\Omega) + \frac{1}{8}\left[1 - \frac{\omega}{\Omega}\right] f\left(\frac{1}{2}a + \frac{1}{2}\omega + \frac{1}{2}\Omega\right) + \frac{1}{8}\left[1 - \frac{\omega}{\Omega}\right] f\left(\frac{1}{2}a - \frac{1}{2}\omega - \frac{1}{2}\Omega\right) + \frac{1}{8}\left[1 + \frac{\omega}{\Omega}\right] f\left(\frac{1}{2}a - \frac{1}{2}\omega + \frac{1}{2}\Omega\right) + \frac{1}{8}\left[1 + \frac{\omega}{\Omega}\right] f\left(\frac{1}{2}a + \frac{1}{2}\omega - \frac{1}{2}\Omega\right) \quad \text{(SE8)}$$

where:

$$a = \frac{g\mu_B B_{hf}}{\hbar} \quad \text{(SE9)}$$

$$\omega = \frac{g\mu_B B_{external}}{\hbar} \quad \text{(SE10)}$$

$$\Omega = \sqrt{a^2 + \omega^2} \quad \text{(SE11)}$$

$$f(x) = \frac{k^2}{k^2 + x^2} \quad \text{(SE12)}$$

Again, B_{hf} is the local hyperfine field, μ_B is Bohr magneton, g is the g-factor, $\hbar$ is the reduced Planck constant. a is the hyperfine coupling constant. $B_{external}$ is the externally applied magnetic field, ω is the Larmor frequency under applied magnetic field. k is the rate constant as discussed in equation (7).

S6 Details of the fitting procedure

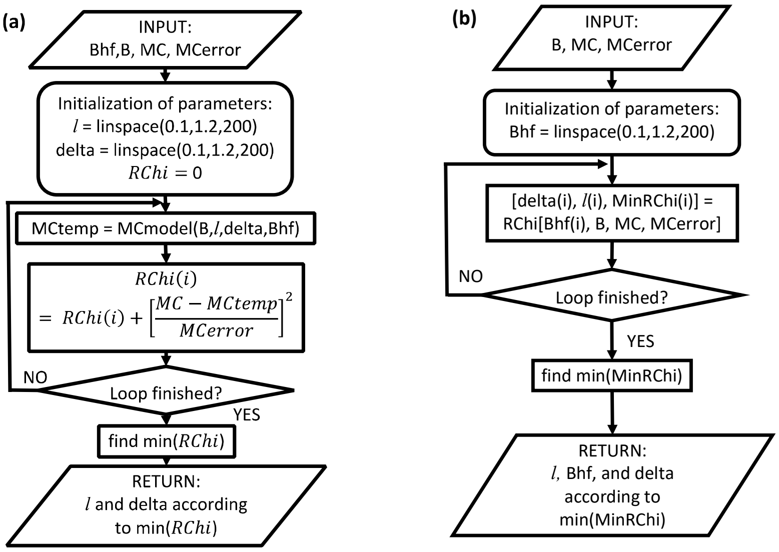

Figure S6. Flow charts of (a) *RChi* function and (b) *RChi3* function algorithms.

The algorithms for fitting the polaron pair model to our experimentally obtained data are shown in Figure S6. The procedure was performed as a short routine using the commercial software Matlab. For the purposed of fitting, l was used instead of k as defined in equation (7). The two parameters are related simply using equation (SE13):

$$l = \frac{k}{a} \quad \text{(SE13)}$$

where k is the rate constant and a is the hyperfine coupling constant as defined using (SE9).

Fitting was performed using two functions, *RChi* and *RChi3*. As shown in Figure S6 (a), *RChi* is the basic function that returns the minimum χ^2_{red} and the optimal l and delta that correspond to k and δ_{TS} ,defined in equations (7) and (8) respectively, using four input parameters: the local hyperfine field, Bhf, raw data B field, B, MC values, MC, and calculated MC standard errors, MCerror. Notably, the function *MCmodel* embedded in function *RChi* is the polaron pair model as described in the main text and in section S6. The function *RChi3* has the function *RChi* embedded and can finally yield the optimal hyperfine field, l and delta that correspond to B_{hfc1}, k and δ_{TS} in equations (4), (7) and (8) respectively with a global minimum χ^2_{redG}. In this way we obtain all three fitting parameters, and also an array of χ^2_{red} values for all individual fitting parameter values attempted. More detailed mathematical descriptions of the fitting procedure and associated calculations appear in the following sections.

Definition of χ^2_{red}

This is defined in equations (SE14) and (SE15).

$$\chi^2 = \sum_1^N \left(\frac{MC(B)_{exp.} - M\ \ (B)_{model}}{\sigma_{exp.}}\right)^2 \qquad \text{(SE14)}$$

$$\chi^2_{red} = \frac{\chi^2}{K} \qquad \text{(SE15)}$$

Where: $MC(B)_{exp.}$ is the experimental data, $MC(B)_{model}$ is the value obtained by the P-P model (equation (8)), $\sigma_{exp.}$ is the error on each experimental data point, N is the number of data points and K the number of degrees of freedom.

In fitting, we have minimised χ^2_{red}, using the approximation $K \approx N$ (given that we are fitting over 375 data points).

Details of χ^2minimisation and optimum fitting parameter selection

The overall minimisation method used is to generate a matrix of χ^2 values using a range of the fitting parameter numerical values (B_{hf}, k and δ_{TS}), over a region of the three dimensional parameter space available. The χ^2 thus generated is then searched numerically to find the minimum global χ^2 value. The set of fitting parameters (B_{hf}, k and δ_{TS}), corresponding to the minimum global χ^2 and corresponding reduced value, $\chi^2_{red,Global}$, are then chosen as the minimisation output.
We do not use an available (or mathematically defined) minimization algorithm, such as gradient descent method used or machine learning techniques.

More specifically:
The function RChi generates a two dimensional matrix of χ^2 values parametric in l and δ_{TS} at a given value of B_{hf}. It does this by running the model to evaluate the MC over all applied magnetic fields and compared to the measured MC (calculating the χ^2). This is done by two nested loops, the outer running over values of l and the inner over values of δ_{TS}. It also selects the minimum χ^2 for that specific value of B_{hf} which it outputs.
The function RChi itself is run within a loop, over different values of B_{hf} , in RChi3 which generates an array of χ^2 values. This array is then searched for the global χ^2 minimum. The reduced $\chi^2_{red,Global}$ is calculated and returned, together with the corresponding set of B_{hf}, k and δ_{TS} values.

Figure S6(a) is a flowchart of the function RChi and we note that the indicated loop consists of two nested loops, over different values of l and δ_{TS}. Figure S6(b) is a flowchart of function RChi3 and the single loop is run over different values of B_{hf}.

Mathematically, individual χ^2 values used in function RChi are defined in equation (SE16).

$$\chi^2{}_{\delta_m,l_n}\left(B_{hf}, l, \delta_{TS}\right) = \sum_{over\ all\ B_{applied}} \left(\frac{MC\left(B_{applied}\right)-Model\left(B_{applied}\right)}{MCError\left(B_{applied}\right)}\right)^2\Bigg|_{B_{hf}} \quad \text{(SE16)}$$

Where $MC\left(B_{applied}\right)$ is the experimentally obtained magnetoconductance at a given applied field and $MCError\left(B_{applied}\right)$ is the experimentally obtained error associated with every specific value of magnetoconductance. $Model\left(B_{applied}\right)$ is the calculated magnetoconductance using the model at specific values of B_{hf}, l, δ_{TS}.
A two dimensional matrix of χ^2 values at a given B_{hf} is generated by the function RChi as shown in equation (SE17)

$$\chi^2{}_{\delta_m,l_n} = \begin{bmatrix} \chi^2{}_{\delta_1,l_1} & \cdots & \chi^2{}_{\delta_1,l_N} \\ \vdots & \ddots & \vdots \\ \chi^2{}_{\delta_N,l_1} & \cdots & \chi^2{}_{\delta_N,l_N} \end{bmatrix} \qquad \text{(SE17)}$$

The minimum value of χ^2 at a given B_{hf} is selected from the $\chi^2{}_{\delta_m,l_n}$ matrix, as shown in equation (SE18), and the corresponding local values of $\delta_{local}, l_{local}$ are noted.

$$\chi^2{}_{\delta_m,l_n}\Big|_{minimum} = \chi^2_{min}\left(B_{hf}\right)\big|_{\delta_{local},l_{local}} \qquad \text{(SE18)}$$

The outputs of the function RChi are used by function RChi3 to construct an array of the χ^2 and associated δ_{TS} and l, over different values of B_{hf} as shown in equation (SE19).

$$\chi^2{}_{min_{Bhf_m}} = \begin{pmatrix} \chi^2_{min}\left(B_{hf1}\right), \delta_{local1}, l_{loca} \\ \vdots \\ \chi^2_{min}\left(B_{hfN}\right), \delta_{localN}, l_{localN} \end{pmatrix} \qquad \text{(SE19)}$$

The minimum value of χ^2 in the array defined by (SE19) corresponds to the global minimum χ^2, as defined in equation (SE20).

$$\chi^2{}_{min_{Bhf_m}}\Big|_{minimum} = \chi^2_{global\ min.}\big|_{\delta_{global},Bhf_{global},l_{global}} \qquad \text{(SE20)}$$

The reduced global $\chi^2_{red,Global}$ is then evaluated using $\chi^2{}_{min_{Bhf_m}}$ and equation (SE15). The corresponding values of B_{hf} and δ_{TS} are reported directly as fitting parameters and the decay rate, k, is calculated using the value for l at minimum χ^2 and equation (SE13).

Additional References:

S1. Konowalczyk, M. Liouville von Neumann simulation by density matrix propagation. https://uk.mathworks.com/matlabcentral/fileexchange/64703-liouville-von-neumann-simulation-by-density-matrix-propagation (2018).
